# A multidimensional normative framework for fine-grained social cognition assessment with the BCS

**DOI:** 10.1186/s40359-026-05124-x

**Published:** 2026-07-07

**Authors:** Arthur Trognon, Nami Koïdé, Natacha Stortini, Coralie Duman, Loann Mahdar-Recorbet, Alizée Poli, Hamza Altakroury

**Affiliations:** 1 CLINICOG, Nancy, France; 2 Faculty of Arts and Humanities Campus, Lorraine University, Nancy, France

**Keywords:** Computational psychology, Social cognition, Facial emotion recognition, Theory of mind, Normative data, Neuropsychological assessment

## Abstract

**Objective:**

Current social cognition assessments often yield global summary scores that obscure clinically meaningful variability across subcomponents. This study proposes a multilevel interpretive framework for the Social Cognition Battery (Batterie de Cognition Sociale, BCS) that enables fine-grained analysis of facial emotion recognition, mental state inference, and theory of mind reasoning.

**Method:**

Analyses were based on partially overlapping normative samples of healthy French-speaking adults (age range: 20–69 years) who completed facial emotion recognition (FER/IDEMO; *n* = 155), Reading the Mind in the Eyes Test (RMET; *n* = 175), and theory of mind (ToM) tasks assessing first-order and second-order belief attribution as well as faux pas recognition (*n* = 129–145; adapted from Baron-Cohen, 2000). A data-driven stratification approach identified optimal normative groupings based on empirically observed age and sex effects. Scoring dimensions included emotion-specific accuracy, intensity-dependent performance profiles, systematic misattribution patterns (confusion matrices), response latencies, and ToM subcomponents by inferential order and cognitive dimension.

**Results:**

Age-related decline emerged for anger and fear recognition (*p* = .001) but not for joy or disgust, which showed ceiling effects. Response times revealed nonlinear age trajectories, with adults aged 50–59 demonstrating longer latencies than those over 60 (*p* < .001). Older adults (60+) exhibited significantly elevated misattribution rates (*p* = .008), whereas a descriptive tendency toward higher anger-disgust confusion in young men did not reach significance after correction for within-participant clustering. Theory of mind performance remained stable across age groups, with significant differentiation across inferential orders (*p* < .001) and cognitive dimensions (*p* < .001).

**Conclusions:**

This multidimensional framework provides normative benchmarks that may enhance clinical utility by enabling identification of emotion-specific performance profiles, detection of processing efficiency variations through response latencies, and characterization of systematic error patterns; thereby extending the interpretive precision of well-validated instruments whose clinical sensitivity has been established across neurological and psychiatric populations.

**Supplementary Information:**

The online version contains supplementary material available at https://doi.org/10.1186/s40359-026-05124-x.

## Background

Social cognition plays a fundamental role in adaptive human functioning, supporting capacities ranging from everyday social exchanges and relationship maintenance to occupational functioning and independent living. Deficits in social cognitive abilities have been documented across a wide spectrum of neurological and psychiatric conditions, including frontotemporal dementia [1–4], schizophrenia [5–7], autism spectrum disorder [8–12], traumatic brain injury [13–15], and various neurodegenerative diseases [16–22]. These impairments carry substantial functional consequences, affecting interpersonal relationships, vocational outcomes, and overall quality of life [23, 24]. The clinical importance of accurately detecting social cognitive deficits (particularly in their early or subtle manifestations) has driven the development of standardized assessment batteries with accompanying normative data.

Although research has consistently demonstrated the importance of social cognition for adaptive functioning and clinical outcomes, the routine assessment of social cognitive abilities remains unevenly implemented across neuropsychological practice. In many clinical settings (particularly in the evaluation of neurodegenerative diseases), social cognition is not yet systematically integrated into standard neuropsychological batteries, owing in part to the limited availability of well-normed instruments, the absence of consensus guidelines on when and how to assess these abilities, and time constraints inherent to clinical evaluation [25, 26]. This gap between established research relevance and actual clinical uptake is compounded by several limitations inherent to current assessment approaches, which further constrain their diagnostic utility and sensitivity to individual differences. Indeed, many widely used instruments yield only global summary scores that obscure clinically meaningful variability across subcomponents of social cognition [25, 26]. For example, a total score on an emotion recognition task does not distinguish between selective impairments for specific emotions (e.g., fear vs. disgust), nor does it capture variations in performance as a function of stimulus difficulty or ambiguity [27, 28]. This distinction is clinically consequential, as different neurological and psychiatric conditions are associated with dissociable emotion-specific impairments. For instance, selective fear recognition deficits have been consistently linked to amygdala damage [29, 30], impaired disgust recognition characterizes Huntington’s disease even at presymptomatic stages [31, 32], and schizophrenia is associated with disproportionate difficulty processing negative emotions with distinct error signatures [33]. Identifying which emotions are selectively affected thus carries diagnostic and mechanistic relevance that aggregate scores cannot provide.

Similarly, aggregate theory of mind scores may mask differential abilities across first-order versus second-order mentalizing, or across distinct cognitive operations such as inference, memory, and cognitive empathy, here defined as the ability to infer the emotional consequences of a social situation for another person, rather than affective empathy in the sense of shared emotional experience [34–36]. Moreover, normative data for social cognition measures remain sparse in many linguistic and cultural contexts, and existing norms often fail to account for demographic variables known to influence performance, including age, sex, and educational attainment [27, 37, 38]. This limitation is particularly pronounced for French-speaking populations, where standardized instruments with adequate normative stratification are scarce.

Beyond these structural and normative limitations, a substantial proportion of the data routinely collected during social cognition assessments remains underexploited in clinical practice. Response times, for instance, provide valuable information about processing efficiency and automaticity [39, 40]. This is particularly relevant in autism spectrum conditions, where response time prolongation has been shown to index effortful compensatory processing despite near-normal accuracy levels [41, 42], and in traumatic brain injury, where slowed emotion processing may persist as a sensitive marker of residual impairment even after accuracy has recovered [43]. Yet those measures are rarely incorporated into normative frameworks or interpretation guidelines [26, 44]. This omission is consequential: processing speed may reveal subtle impairments that remain undetected when only accuracy is considered [45, 46], particularly in high-functioning individuals who achieve ceiling-level accuracy through compensatory effortful processing [47–49]. Similarly, error patterns (i.e. the systematic confusion between specific emotion pairs or mental state categories) are typically recorded but seldom formalized into standardized scoring systems [28, 50]. Such patterns carry potential diagnostic significance, as they may distinguish between qualitatively different impairment mechanisms, such as perceptual deficits versus conceptual confusion [30, 51]. For instance, patients with schizophrenia tend to exhibit systematic over-attribution of anger and threat-related emotions across diverse facial expressions [50], whereas patients with amygdala lesions show selective confusion between fear and surprise while other emotion pairs remain intact [43]. These distinct error signatures may inform differential diagnosis and guide targeted intervention, yet their clinical exploitation currently depends on informal clinical judgment rather than empirically derived norms, limiting both reliability and comparability across assessments [25, 52].

The Social Cognition Battery (Batterie de Cognition Sociale, BCS; [53–58]) illustrates both the strengths and the shortcomings of current normative practices in social cognition assessment. This French-language battery provides a comprehensive evaluation encompassing facial emotion recognition (the IDEMO task ; Facial Emotion Recognition/FET ; adapted from [59]), mental state inference from eye regions (an adaptation of the Reading the Mind in the Eyes Test; [60]), and theory of mind reasoning across first-order, second-order, and faux pas domains (adapted from [61]).

Importantly, these three subtests map onto the established distinction between cognitive and affective components of theory of mind [36, 62, 63]. Cognitive ToM refers to the ability to infer others’ beliefs, intentions, and knowledge states, whereas affective ToM involves inferring others’ emotional experiences [36]. Within this framework, the first-order and second-order belief attribution tasks primarily engage cognitive ToM, while the RMET is commonly considered a measure of affective ToM, requiring the decoding of complex mental states (including emotional states) from perceptual cues [60], although its precise theoretical status remains debated, with some authors characterizing it as an advanced emotion recognition task rather than a ToM measure per se [64, 65]. The faux pas tasks occupy a hybrid position, requiring both cognitive inference about the transgressor’s false belief and affective inference about the emotional consequences for the victim, a distinction directly captured by the empathy dimension in the present scoring framework. By treating these subtests as separate measures rather than aggregating them into a single social cognition composite, the proposed framework preserves the cognitive–affective distinction and enables clinicians to identify dissociations between these components.

The battery generates rich, multidimensional data: beyond simple accuracy totals, the IDEMO/FET task yields emotion-specific recognition rates, performance profiles across varying levels of emotional intensity (from subtle/ambiguous to prototypical expressions), systematic patterns of emotional confusion (misattributions), and response latencies. Similarly, the theory of mind tasks permit scoring not only by inferential order but also by underlying cognitive dimension (memory, inference, false belief attribution, etc.).

Yet the published normative data for the BCS do not exploit this multidimensional richness. Current norms express individual performance as a distance in standard deviations from the group mean on total raw scores. This approach rests on the assumption of normally distributed performance [66, 67] and offers limited precision for identifying clinically significant deviations at the extremes of the distribution [68, 69]. Standard deviation-based norms may inadequately capture performance variability in measures prone to ceiling or floor effects [70, 71], particularly in social cognition assessment [72] where certain emotions (e.g. joy) are recognized near-perfectly while others (e.g. fear, anger) show greater individual differences [73, 74]. Percentile-based norms offer several advantages in this context: they make no distributional assumptions [68, 75], provide intuitive clinical interpretation [69, 76], and allow precise localization of individual performance within the normative distribution regardless of skewness or kurtosis [67, 75]. Furthermore, accumulating evidence shows that demographic effects on social cognition are heterogeneous: age-related decline is well-documented for recognition of certain emotions (particularly anger, fear, and sadness) but not others such as disgust [77–80]; sex differences, though consistently favoring females, vary in magnitude by task characteristics, stimulus modality, and specific emotion [81–84]; and theory of mind abilities, while showing age-related decline, may be partially preserved or more strongly mediated by executive function demands than emotion recognition per se [85–88]. A uniform stratification strategy cannot accommodate this heterogeneity and may result in norms that are simultaneously overstratified for some measures (wasting statistical power) and understratified for others (losing clinical sensitivity).

The present study thus proposes a novel interpretive methodology for the BCS that moves beyond global summary scores to enable fine-grained, multidimensional analysis of social cognitive performance. This approach is predicated on the recognition that clinically meaningful variability often resides at the level of specific subcomponents, and that aggregate scores may mask dissociated profiles with distinct etiological, prognostic, and rehabilitative implications.

Specifically, we propose a multilevel scoring and interpretation framework encompassing: (a) emotion-specific accuracy scores and intensity-dependent accuracy profiles on the FER task; (b) systematic quantification of emotional shifting (misattribution) patterns using confusion matrices; (c) response time analyses stratified by emotion and by intensity level; (d) accuracy and response time norms for the RMET; and (e) order-based (first-order, second-order, faux pas) and dimension-based (memory, inference, false belief, factual, detection, identification, non-intentionality, empathy) scoring frameworks for the ToM subtest.

To support this interpretive methodology, we establish empirically derived normative data for each scoring dimension, employing a data-driven stratification approach that identifies optimal groupings based on age and sex effects rather than imposing a priori demographic categories. This strategy maximizes statistical power while preserving clinically meaningful distinctions, ensuring that normative groups reflect genuine performance differences rather than arbitrary partitions.

The resulting interpretive framework is intended to enhance the clinical utility of the BCS by providing clinicians with normative benchmarks to: (a) detect performance falling below expected levels for specific emotions or cognitive operations; (b) characterize the influence of stimulus ambiguity on recognition performance; (c) identify response time patterns that may reflect compensatory processing; (d) interpret systematic error patterns that may inform a more fine-grained understanding of social cognitive difficulties; and (e) distinguish between first-order and higher-order mentalizing performance. We anticipate that this multilevel approach has the potential to increase diagnostic sensitivity, support individualized rehabilitation planning, and facilitate more precise outcome monitoring in clinical populations once validated in relevant diagnostic groups.

## Method

### Dataset

The present study used normative data collected as part of the validation of the Social Cognition Battery (Batterie de Cognition Sociale, BCS), a French-language instrument developed for the assessment of social cognitive functions in neurological populations [53]. The normative sample was originally collected by Ehrlé et al. (2011) as part of the BCS validation. The original authors describe recruiting healthy French-speaking adults from the general population, with participants screened for neurological and psychiatric history. However, because the present study constitutes a secondary analysis of this publicly available dataset, the specific screening procedures (e.g., structured clinical interview, self-report questionnaire, or clinician-administered checklist) are not fully detailed in the original publication.

Importantly, the BCS normative dataset does not derive from a single fixed cohort administered all subtests. Rather, the original validation employed partially overlapping samples across subtests, with independent recruitment and exclusion procedures applied to each task. The resulting normative sample sizes therefore vary by subtest: the Facial Emotion Recognition task (FER/IDEMO) retained 155 participants (from an initial pool of 156, with 1 exclusion based on a -3 SD criterion on total accuracy); the Reading the Mind in the Eyes Test (RMET) retained 175 participants (from 175 initially recruited, with no exclusions); and the Theory of Mind tasks retained 145 participants (the official manual states that : “from 149 initially recruited, with 3 exclusions based on a -3 SD criterion on total score”). For the present analyses, additional exclusions were applied within specific scoring frameworks (e.g., participants with missing data on any ToM item were further excluded from dimension-based scoring, yielding a final analytic sample of 129 for that analysis).

Women had a mean age of 41.96 years (SD = 12.95; range: 21–67) and a mean of 12.31 years of formal education (SD = 3.04; range: 9–17). Men had a mean age of 39.12 years (SD = 14.23; range: 20–69) and a mean of 12.89 years of formal education (SD = 2.88; range: 9–17). Participants were originally stratified into five age groups (20–29, 30–39, 40–49, 50–59, and 60–69 years) and three educational levels (below baccalaureate, baccalaureate, above baccalaureate) for normative purposes. Sample sizes varied slightly across subtests due to additional exclusions based on task-specific outlier criteria, as detailed in each analysis section.

### The Social Cognition Battery (BCS)

The Social Cognition Battery (Batterie Cognition Sociale, BCS) is a standardized French-language instrument designed to assess core components of social cognition, including facial emotion recognition, mental state attribution from eye gaze, and theory of mind reasoning. The battery comprises nine subtests administered in a fixed order: (1) Facial Emotion Identification (2), Facial Emotion Discrimination (3), Expressive Intensity Judgment (4), Reading the Mind in the Eyes (5), Gender Identification (6), Humor Identification (7), Social Situations Task (8), Moral and Conventional Judgments, and (9) Theory of Mind Tasks (first-order, second-order, and faux pas). The present study focused on three subtests that yield particularly rich multidimensional data amenable to the proposed interpretive framework: Facial Emotion Identification (IDEMO), Reading the Mind in the Eyes (Langage des Yeux), and Theory of Mind Tasks.

#### Facial Emotion Recognition Task (FER/IDEMO)

The FER task assesses the ability to identify six basic emotions (anger [A], disgust [D], joy [J], fear [F], surprise [Surp], and sadness [Sad]) from facial expressions. The task comprises 60 stimuli (10 per emotion), each displaying a face transitioning from a neutral expression to a target emotion. Emotional intensity varies parametrically across 10 levels (coded 10–19 in raw data), with lower values corresponding to more subtle, ambiguous expressions and higher values to more prototypical, easily recognizable displays. Participants select the emotion they perceive from a six-alternative forced-choice response format. Both accuracy (number of correct identifications) and response time (in milliseconds) are recorded for each trial.

#### Reading the Mind in the Eyes Test (RMET)

The French adaptation of the Reading the Mind in the Eyes Test [60] was administered to assess the ability to infer complex mental states from photographs of the eye region. Participants viewed 36 black-and-white photographs depicting the eye region of adult faces and selected the most appropriate mental state descriptor from four alternatives. Accuracy scores range from 0 to 36, with higher scores indicating better mental state recognition. Response times were recorded for each trial.

#### Theory of Mind Tasks (ToM)

The Theory of Mind subtest assesses the ability to attribute mental states to others across three domains of increasing complexity: first-order belief attribution (understanding that another person can hold a belief different from reality), second-order belief attribution (understanding that one person can hold a belief about another person’s belief), and faux pas recognition (detecting social transgressions resulting from false beliefs about others’ mental states). The subtest comprises 6 items distributed across the three domains: first-order tasks (10 items), second-order tasks (7 items), and faux pas tasks (14 items). For each scenario, participants listen to a short story and answer questions probing different cognitive components: memory (story recall), inference (mental state attribution), false belief understanding, factual comprehension (non-mental story content), faux pas detection, identification of the transgressor, recognition of non-intentionality, and empathy (inferring emotional consequences). Accuracy is recorded for each sub-item, yielding both order-based scores (first-order, second-order, faux pas, and total) and dimension-based scores across the eight cognitive components.

### Data processing and scoring

Data processing and scoring were performed using custom scripts in Python 3.10 (pandas, numpy, statsmodels libraries) and R 4.2 (psych, lavaan, dplyr, readxl packages). All scripts are available upon reasonable request from the corresponding author.

#### FER(IDEMO) accuracy scores

For each participant, accuracy was computed at three levels of analysis. First, emotion-specific accuracy scores were calculated as the number of correct identifications out of 10 trials per emotion category, yielding scores ranging from 0 to 10 for each of the six emotions and a total score ranging from 0 to 60. Next, intensity-level accuracy scores were computed as the number of correct identifications across the six emotions at each intensity level [10–19], with scores ranging from 0 to 6 per level. Finally, proportional accuracy scores (ranging from 0 to 1) were derived by dividing raw scores by the number of items at each level.

#### FER(IDEMO) emotional shifting

Emotional shifting (misattribution) patterns were quantified by computing confusion matrices for each participant. For each trial where the participant’s response differed from the target emotion, the error was coded as a shift from the expected emotion to the given response. Shift counts were aggregated across all trials to generate 30 shift variables (6 emotions × 5 possible incorrect responses) per participant. This approach captures systematic confusion patterns (e.g., fear misidentified as surprise) that may reflect underlying processing biases or perceptual similarities between emotion categories.

#### FER(IDEMO) response times

Mean response times were computed separately for each emotion category (averaging across the 10 trials per emotion) and for each intensity level (averaging across the 6 emotions per intensity level). Trials with missing response time values (i.e., not recorded due to technical failure) or with response times equal to zero (indicating a recording error) were excluded from averaging. No additional outlier trimming or data transformation was applied to response time distributions.

#### Reading the mind in the eyes test

As the RMET uses a four-alternative forced-choice format in which participants select one predefined mental state descriptor per item, raw responses were preprocessed by mapping numeric response codes to their corresponding mental state labels using a pairing table. Column headers were then renamed to reflect the correct mental state for each item. These string operations served exclusively for data organization and readability purposes and did not affect the scoring procedure. Accuracy was computed by comparing each participant’s response (converted to lowercase) to the expected label derived from the item name (with numeric suffixes removed, e.g., “Rêveur.1” → “rêveur”). The total accuracy score was calculated as the sum of correct responses across the 36 items, yielding scores ranging from 0 to 36. Mean response time (in milliseconds) was computed by averaging response latencies across all items for each participant. Trials with missing response time values (i.e., not recorded due to technical failure) or with response times equal to zero (indicating a recording error) were excluded from averaging. No additional outlier removal procedure or data transformation was applied to response time distributions. Response time values containing commas as decimal separators were converted to standard numeric format prior to analysis.

#### Theory of mind

Raw item-level responses were organized according to three task domains identified by column prefixes: first-order belief tasks (FO_), second-order belief tasks (SO_), and faux pas recognition tasks (FP_). An additional trimming was applied: participants with missing data on any ToM item were excluded from analysis (16 participants removed, 129 retained). This complete-case approach was adopted because the ToM subtest comprises items that are conceptually interdependent within each scenario (e.g., memory, inference, and false belief questions all relate to the same story). Imputing individual item responses within such a structure risks generating internally inconsistent response profiles (e.g., a participant scored as correctly attributing a false belief but failing the memory question for the same story), which would distort both order-based and dimension-based scoring. Complete-case analysis was therefore retained as the most conservative and transparent approach for establishing normative benchmarks, though we acknowledge that this decision reduced statistical power and may have introduced selection bias if missingness was systematically related to participant characteristics. Two complementary scoring frameworks were applied. For order-based scoring, total scores were computed by summing correct responses within each domain: first-order (maximum = 10), second-order (maximum = 7), and faux pas (maximum = 14), yielding a total score ranging from 0 to 31. For dimension-based scoring, items were classified according to eight cognitive components: memory (story recall), inference (mental state attribution), false belief (understanding of mistaken beliefs), factual comprehension (non-mental story content), detection (identifying the presence of a faux pas), identification (specifying the transgressor), non-intentionality recognition (understanding the unintended nature of the offense), and empathy (inferring emotional consequences). Dimension-specific scores were computed by summing correct responses across all items tapping each cognitive component, regardless of task domain. Cross-tabulated scores (dimension × domain) were also computed where applicable.

#### Normative group determination

A data-driven approach was employed to determine optimal normative stratification. Preliminary analyses examined the effects of age (treated as a categorical variable with five levels: 20–29, 30–39, 40–49, 50–59, and 60 + years) and sex on each outcome measure. For continuous outcomes (accuracy scores, response times), factorial ANOVAs with Type II sums of squares were conducted, with complementary ANCOVAs employed when examining linear trends across ordinal predictors (e.g., emotional intensity treated as a continuous covariate). For all mixed-model ANOVAs involving within-subjects factors (emotion category, intensity level, order of inference, or cognitive dimension), Mauchly’s test of sphericity was computed; when the sphericity assumption was violated (*p* < .05), degrees of freedom were corrected using the Greenhouse-Geisser epsilon adjustment, and corrected F-values, degrees of freedom, and p-values are reported. For count data (emotional shifting frequencies), each participant contributed one observation per emotion pair (6 target emotions × 5 possible incorrect responses = 30 observations per individual). To account for this within-participant dependency structure, generalized estimating equations (GEE) were fitted with participant specified as the clustering variable and an exchangeable working correlation structure, ensuring that standard errors were not underestimated due to non-independence of observations. Robust sandwich standard errors were used to provide valid inference regardless of potential misspecification of the working correlation structure. Where significant age × sex interactions emerged, post-hoc pairwise comparisons (Tukey’s Honestly Significant Difference test for ANOVA; pairwise contrasts with Bonferroni correction for GEEs) were conducted to identify which subgroups differed significantly. Effect sizes were reported for all analyses to complement significance testing and inform normative stratification decisions. For ANOVAs and ANCOVAs, partial eta-squared (ηp²) with 90% confidence intervals [89] and partial omega-squared (ωp²) were computed for all main effects and interactions. Effect magnitudes were interpreted using Cohen’s conventional benchmarks [90]: small (ηp² ≈ 0.01), medium (ηp² ≈ 0.06), and large (ηp² ≈ 0.14). For post-hoc pairwise comparisons (Tukey’s Honestly Significant Difference test), Cohen’s d with 95% confidence intervals was computed to quantify the magnitude of group differences. For GEEs, incidence rate ratios (IRR) with 95% confidence intervals were derived by exponentiating regression coefficients, providing an interpretable metric of the multiplicative change in outcome frequency relative to the reference category. Normative groups were established by combining subgroups that did not differ statistically and that exhibited negligible effect sizes (d < 0.20 or ηp² < 0.01). When these quantitative criteria conflicted with practical constraints (such as insufficient cell sizes to establish stable norms, or subgroup differences driven by a single sex × age cell) clinical practicality and normative stability were prioritized, with the rationale for each departure documented in the corresponding results section.

## Results

### Facial emotion recognition

#### Accuracy for emotions

Data for accuracy scores on the FER task are presented in Table 1. Overall, results revealed ceiling effects for joy and disgust recognition, age-related decline for anger and fear identification, and minimal sex differences across all emotions.

**Table 1:** Accuracy scores for the FER task across three age groups

Group	18–59	60+
n	134	21
Men	52	13
Women	82	8
Age	37.26 ± 10.95	63.05 ± 2.92
Anger	7.88 ± 2.49	6.24 ± 2.93
Disgust	9.48 ± 1.25	9.48 ± 0.81
Joy	9.94 ± 0.34	10.00 ± 0.00
Fear	8.54 ± 1.95	7.00 ± 2.55
Surprise	9.46 ± 0.85	8.81 ± 1.89
Sadness	8.49 ± 2.20	7.90 ± 1.95
Total	53.78 ± 5.44	49.43 ± 5.46

A total of 155 healthy French-speaking participants were stratified into younger adults (18–59 years; *n* = 134) and older adults (60 + years; *n* = 21). Accuracy scores reflect the number of correctly identified emotions out of 10 trials per emotion category, with total scores ranging from 0 to 60. Joy and disgust showed ceiling effects across all age groups, whereas anger and fear demonstrated greater variability and age-related decline. Values are presented as mean ± SD. In group labels, numeric ranges (e.g., 18–29) indicate age in years. Percentile-based normative data are provided in Additional File 1

A three-way mixed-model ANOVA was conducted with age group (18–29, 30–39, 40–49, 50–59, 60+) and sex as between-subjects factors and emotion (anger, disgust, joy, fear, surprise, sadness) as a within-subjects factor. Type II sums of squares were computed using the statsmodels library in Python. Mauchly’s test indicated a violation of sphericity for the emotion factor, W = 0.216, χ² (14) = 233.18, *p* < .001; degrees of freedom for all within-subjects effects were therefore corrected using the Greenhouse-Geisser adjustment (ε = 0.705). Results revealed a significant main effect of emotion [F(3.52, 613.06) = 38.99, *p* < .001, ηp² = 0.183, 90% CI [0.149, 0.218], ωp² = 0.170], indicating large differences in recognition accuracy across emotion categories. A significant main effect of age group was also observed [F(4, 870) = 4.99, *p* = .001, ηp² = 0.022, 90% CI [0.006, 0.038], ωp² = 0.017], reflecting a small but reliable age-related effect on overall accuracy. The age × emotion interaction remained significant after correction [F(14.09, 613.06) = 1.80, *p* = .035, ηp² = 0.040, 90% CI [0.004, 0.041], ωp² = 0.017], suggesting that age-related decline was not uniform across emotions. The main effect of sex approached but did not reach significance [F(1, 870) = 3.65, *p* = .056, ηp² = 0.004, ωp² = 0.003], nor were the age × sex [F(4, 870) = 0.82, *p* = .511, ηp² = 0.004, ωp² < 0.001], sex × emotion [F(3.52, 613.06) = 1.02, *p* = .391, ηp² = 0.006, ωp² < 0.001], or three-way interactions [F(14.09, 613.06) = 0.87, *p* = .589, ηp² = 0.020, ωp² < 0.001] significant. Post-hoc Tukey HSD comparisons indicated that the 60 + group performed significantly lower than the 18–29 (*p* = .001, d = 0.42, 95% CI [0.20, 0.64]), 30–39 (*p* = .008, d = 0.34, 95% CI [0.13, 0.55]), and 40–49 (*p* = .004, d = 0.41, 95% CI [0.17, 0.65]) groups, all reflecting small effect sizes. No significant differences emerged among the four younger age groups (all d < 0.14, all *p* > .09). The overall sex difference, though non-significant, corresponded to a negligible effect size (d = 0.14, 95% CI [0.01, 0.27]). Based on this pattern, normative data were stratified into two age groups (< 60 years vs. ≥ 60 years).

Across both age groups, accuracy was highest for joy (ranging from 9.94 to 10.00) and disgust (9.48 in both groups), indicating ceiling effects for these emotions. In contrast, recognition of fear and anger showed greater variability and declined with age, with mean accuracy scores for anger decreasing from 7.88 (SD = 2.49) in younger adults to 6.24 (SD = 2.93) in older adults, and fear accuracy decreasing from 8.54 (SD = 1.95) to 7.00 (SD = 2.55) across the same comparison. Total accuracy scores followed a similar pattern, with mean scores of 53.78 (SD = 5.44) and 49.43 (SD = 5.46) for the younger and older adult groups, respectively. Sex differences were minimal across all emotions and age groups.

#### Accuracy by intensity level

Data for accuracy scores across intensity levels on the FER (IDEMO) task are presented in Table 2. Overall, results revealed a consistent positive relationship between emotional intensity and recognition accuracy and an age-related decline that was consistent across intensity levels.

**Table 2:** Accuracy scores relative to emotional intensities across the FER (IDEMO) task

Group	18–59	60+
n	134	21
Men (age)	52 (33.85 ± 9.61)	13 (62.69 ± 3.01)
Women (age)	82 (39.43 ± 11.24)	8 (63.62 ± 2.88)
Accuracy(1)	4.93 ± 0.88	4.48 ± 0.81
Accuracy(2)	5.34 ± 0.77	4.86 ± 0.96
Accuracy(3)	5.30 ± 0.82	5.05 ± 1.02
Accuracy(4)	5.37 ± 0.84	4.86 ± 0.85
Accuracy(5)	5.38 ± 0.84	5.14 ± 0.91
Accuracy(6)	5.48 ± 0.85	4.90 ± 1.00
Accuracy(7)	5.42 ± 0.79	5.05 ± 0.86
Accuracy(8)	5.40 ± 0.78	4.81 ± 0.93
Accuracy(9)	5.56 ± 0.69	5.10 ± 0.83
Accuracy(10)	5.60 ± 0.66	5.19 ± 0.75

Scores were computed across ten intensity levels, with higher levels (1–10, corresponding to raw intensity values 10–19) reflecting increasingly explicit emotional expressions. Based on preliminary analyses revealing a significant main effect of age but no significant age × sex interaction on intensity recognition, two normative groups were established. The first group comprised adults aged 18–59 years (*n* = 134; men: *n* = 52, mean age = 33.85 ± 9.61 years; women: *n* = 82, mean age = 39.43 ± 11.24 years). The second group included older adults aged 60 years and above (60+; *n* = 21; men: *n* = 13, mean age = 62.69 ± 3.01 years; women: *n* = 8, mean age = 63.62 ± 2.88 years). A consistent pattern emerged across groups: accuracy increased with emotional intensity. Accuracy scores for each intensity level range from 0 to 6. Data are presented as mean ± standard deviation. Numeric ranges (e.g., 18–59) indicate age in years; values in parentheses denote age in months. Percentile-based normative data are provided in Additional File 2

A three-way mixed-model ANOVA examined the effects of age group (five levels: 18–29, 30–39, 40–49, 50–59, 60+), sex, and intensity level (10 levels, treated categorically) on accuracy scores. Mauchly’s test indicated a violation of sphericity for the intensity factor, W = 0.628, χ² (44) = 70.28, *p* = .007; degrees of freedom for all within-subjects effects were therefore corrected using the Greenhouse-Geisser adjustment (ε = 0.914). Results revealed significant main effects of age group [F(4, 1450) = 13.55, *p* < .001, ηp² = 0.036, 90% CI [0.017, 0.051], ωp² = 0.031], sex [F(1, 1450) = 9.92, *p* = .002, ηp² = 0.007, 90% CI [0.001, 0.016], ωp² = 0.006], and intensity [F(8.23, 1325.19) = 7.75, *p* < .001, ηp² = 0.046, 90% CI [0.023, 0.059], ωp² = 0.038]. The age and intensity effects were small, while the sex effect was negligible in magnitude. The age × sex interaction approached but did not reach significance [F(4, 1450) = 2.24, *p* = .063, ηp² = 0.006, ωp² = 0.003], whereas the age × intensity [F(32.90, 1325.19) = 0.51, *p* = .990, ηp² = 0.013, ωp² < 0.001], sex × intensity [F(8.23, 1325.19) = 0.67, *p* = .726, ηp² = 0.004, ωp² < 0.001], and three-way interactions [F(32.90, 1325.19) = 0.48, *p* = .995, ηp² = 0.012, ωp² < 0.001] were not significant. A complementary ANCOVA treating intensity as a continuous covariate confirmed significant effects of age group [F(4, 1539) = 13.77, *p* < .001, ηp² = 0.035, 90% CI [0.016, 0.049], ωp² = 0.032], sex [F(1, 1539) = 10.08, *p* = .002, ηp² = 0.007, 90% CI [0.001, 0.015], ωp² = 0.006], a linear effect of intensity [F(1, 1539) = 47.23, *p* < .001, ηp² = 0.030, 90% CI [0.016, 0.045], ωp² = 0.029], and a marginally significant age × sex interaction [F(4, 1539) = 2.27, *p* = .060, ηp² = 0.006, ωp² = 0.003].

Post-hoc Tukey HSD tests on age groups indicated that older adults (60+) performed significantly worse than all younger groups: versus 18–29 (*p* < .001, d = 0.57, 95% CI [0.40, 0.74]), 30–39 (*p* < .001, d = 0.50, 95% CI [0.33, 0.66]), 40–49 (*p* < .001, d = 0.60, 95% CI [0.41, 0.79]), and 50–59 (*p* < .001, d = 0.39, 95% CI [0.20, 0.57]), reflecting small to medium effect sizes. No significant differences emerged among the four younger age groups (all d < 0.18, all *p* > .14). Post-hoc comparisons on sex revealed that women outperformed men overall (mean difference = 0.16, *p* < .001, d = 0.19, 95% CI [0.09, 0.29]), a negligible effect size. Exploratory post-hoc analyses on the ten age × sex subgroups indicated that young women (F_18–29) performed significantly better than men aged 18–29 (d = 0.39) and 30–39 (d = 0.35), women aged 50–59 (d = 0.40), and all participants aged 60+ (d = 0.74–0.86), all adjusted *p* < .05. However, young women did not significantly differ from women aged 30–39 or 40–49, nor from men aged 40–49 or 50–59 (all adjusted *p* > .25, all d < 0.30).

Given the absence of a significant age × sex interaction and the post-hoc pattern, two normative groups were established based primarily on age: adults under 60 years (18–59; *n* = 134) and older adults aged 60 years and above (*n* = 21), with sex considered as a potential covariate for score interpretation.

Adults aged 18–59 demonstrated consistent performance across intensity levels, with mean scores progressively increasing from 4.93 (SD = 0.88) at intensity level 10 to 5.34 (SD = 0.77) at level 11, 5.37 (SD = 0.84) at level 13, 5.48 (SD = 0.85) at level 15, and reaching 5.60 (SD = 0.66) at level 19. This pattern indicates a stable linear improvement in recognition accuracy as emotional expressions became more explicit.

In contrast, older adults exhibited lower accuracy overall, with a notable floor effect at lower intensity levels. At intensity level 10, older adults scored 4.48 (SD = 0.81), representing a 0.45-point deficit compared to younger adults. Unlike the younger group, performance in older adults did not increase as consistently with intensity: scores fluctuated between levels, reaching 4.86 (SD = 0.96) at level 11, 5.05 (SD = 1.02) at level 12, decreasing to 4.86 (SD = 0.85) at level 13, and achieving only 5.19 (SD = 0.75) at level 19. Notably, variance was generally higher in the older adult group, particularly at intermediate intensity levels (e.g., SD = 1.02 at level 12; SD = 1.00 at level 15), suggesting greater heterogeneity in the ability to recognize moderately expressed emotions among older individuals. The age-related performance gap remained present across all intensity levels, consistent with the non-significant age × intensity interaction.

#### Emotion shifting

**Fig. 1:**
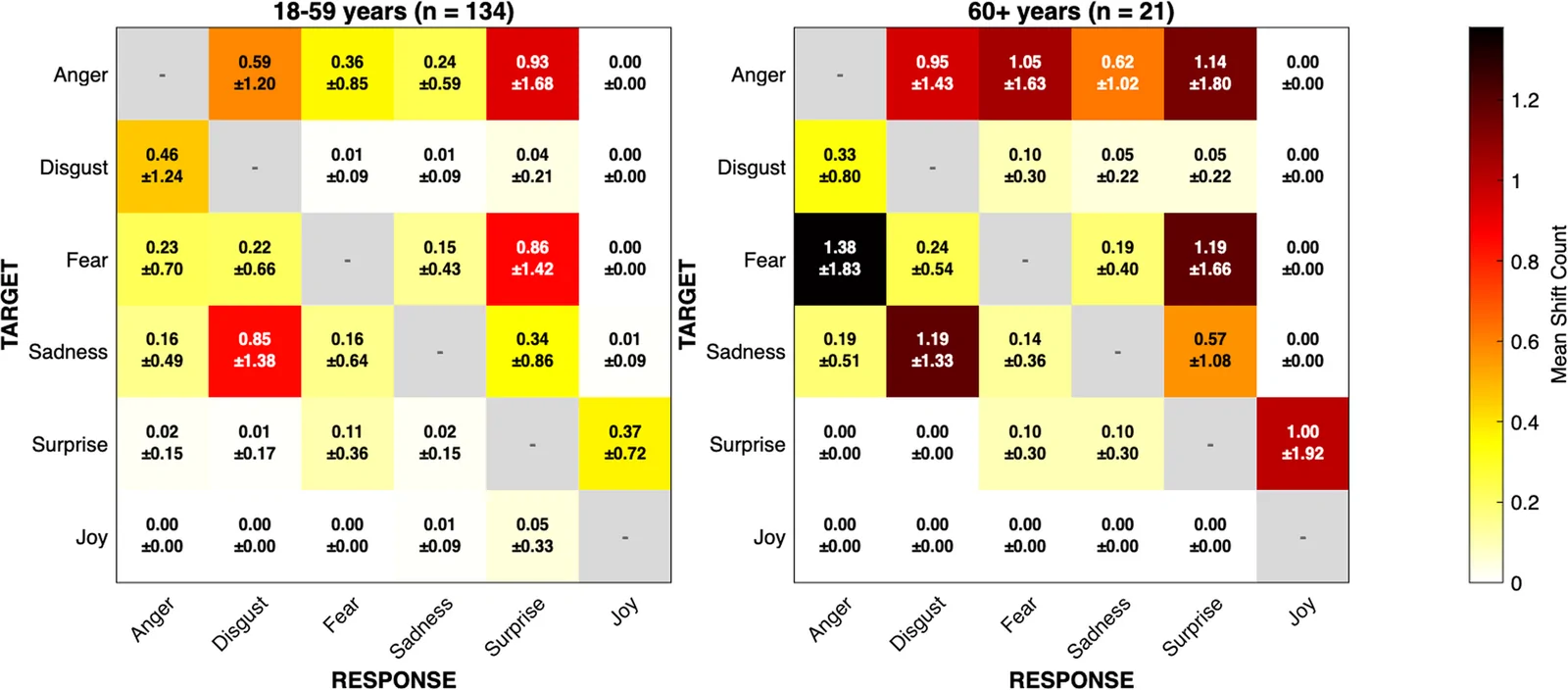
Emotional shifting patterns across sex and age groups on the FER (IDEMO) task. Confusion matrices display the mean frequency (± SD) with which each target emotion (rows) was erroneously identified as another emotion (columns), with diagonal cells (gray) representing correct identifications. Color intensity reflects misattribution frequency, with warmer colors indicating higher error rates. The left panel shows adults aged 18–59 years (*n* = 134; 52 men, 82 women; mean age = 37.26 ± 10.95), and the right panel shows older adults aged 60 years and above (*n* = 21; 13 men, 8 women; mean age = 63.05 ± 2.92). Across both groups, sadness was most frequently confused with disgust, and fear with surprise. Older adults demonstrated markedly elevated misattribution of fear as anger (1.38 ± 1.83 vs. 0.23 ± 0.70) and of surprise as joy (1.00 ± 1.92 vs. 0.37 ± 0.72), patterns largely absent or minimal in younger adults. Joy recognition remained virtually error-free in both groups. Percentile-based normative data are provided in Additional File 3

Data for emotional misattribution patterns on the FER task are presented in Fig. 1. Overall, results revealed systematic confusion between sadness and disgust across all groups, frequent misattribution of fear as surprise that intensified with age, and markedly higher bidirectional confusion between anger and disgust in young men compared to young women. Older adults additionally exhibited elevated fear-anger confusion and increased surprise-joy misattributions.

Given the count nature of shift data and the repeated-measures structure (each participant contributing one observation per emotion pair, yielding 30 observations per individual), a generalized estimating equation (GEE) approach was adopted to account for within-participant dependency. An exchangeable working correlation structure was specified, with robust sandwich standard errors ensuring valid inference. A Poisson family was initially fitted; however, evidence of overdispersion led to the adoption of a negative binomial family. The final model included sex, age group (five levels: 18–29, 30–39, 40–49, 50–59, 60+), their interaction, expected emotion, and given emotion as predictors. Effect sizes are reported as incidence rate ratios (IRR) with 95% confidence intervals.

Results revealed a marginal main effect of sex [b = 0.69, z = 1.77, *p* = .077, IRR = 1.99, 95% CI [0.93, 4.25]], suggesting a tendency for men in the youngest age group (18–29) to commit approximately twice as many misattributions as women, though this effect did not reach conventional significance after accounting for within-participant clustering. Among age group effects, only the 60 + group differed significantly from the 18–29 reference group [b = 0.97, z = 2.64, *p* = .008, IRR = 2.65, 95% CI [1.29, 5.45]], indicating that older adults committed approximately 2.6 times more misattributions. The 50–59 group showed a marginal trend toward elevated error rates [b = 0.67, z = 1.73, *p* = .084, IRR = 1.95, 95% CI [0.91, 4.15]], whereas the 30–39 [b = 0.48, z = 1.35, *p* = .179, IRR = 1.62, 95% CI [0.80, 3.29]] and 40–49 [b = 0.38, z = 1.01, *p* = .310, IRR = 1.47, 95% CI [0.70, 3.07]] groups did not differ significantly. No sex × age interactions reached significance (all *p* > .12), indicating that the attenuation of sex differences with age, though descriptively apparent, could not be statistically confirmed with appropriate correction for within-participant dependency.

Emotion-specific effects were robust and remained highly significant after clustering correction. Compared to anger (reference), misattribution rates were substantially lower when the expected emotion was joy [b = − 3.98, z = − 7.56, *p* < .001, IRR = 0.02, 95% CI [0.01, 0.05]], disgust [b = − 1.43, z = − 7.41, *p* < .001, IRR = 0.24, 95% CI [0.16, 0.35]], sadness [b = − 1.05, z = − 6.57, *p* < .001, IRR = 0.35, 95% CI [0.26, 0.48]], surprise [b = − 0.53, z = − 4.31, *p* < .001, IRR = 0.59, 95% CI [0.46, 0.75]], or fear [b = − 0.47, z = − 4.10, *p* < .001, IRR = 0.62, 95% CI [0.50, 0.78]]. Regarding given emotions, compared to anger, misattributions toward sadness were 64% more frequent [b = 0.49, z = 2.90, *p* = .004, IRR = 1.64, 95% CI [1.17, 2.28]], whereas misattributions toward joy [b = − 1.18, z = − 6.06, *p* < .001, IRR = 0.31, 95% CI [0.21, 0.45]], surprise [b = − 0.97, z = − 4.99, *p* < .001, IRR = 0.38, 95% CI [0.26, 0.56]], and fear [b = − 0.56, z = − 2.75, *p* = .006, IRR = 0.57, 95% CI [0.38, 0.85]] were less frequent; disgust did not differ significantly from anger [b = 0.18, z = 1.29, *p* = .196, IRR = 1.20, 95% CI [0.91, 1.58]].

Based on these findings, normative stratification for emotional shifting followed the same data-driven criteria applied to other FER measures. The GEE analysis confirmed a significant age effect only for the 60 + group (*p*=.008, IRR = 2.65), whereas no other age group differed significantly from the reference. The main effect of sex did not reach significance (*p*=.077), and no sex × age interactions were confirmed (all *p*>.12). Although the IRR for sex (1.99) suggests a non-negligible tendency toward higher misattribution rates in young men, the absence of statistical confirmation and of significant interactions does not meet the criteria for establishing sex-based normative groups. Accordingly, two normative groups were established: adults aged 18–59 years (*n* = 134) and older adults aged 60 years and above (*n* = 21), with sexes combined. Clinicians should note that young men may exhibit somewhat elevated misattribution rates, particularly for bidirectional anger–disgust confusion, though this tendency requires confirmation in larger samples.

Across both normative groups, joy demonstrated near-perfect recognition with virtually no misattributions, confirming a ceiling effect for this emotion (Fig. 1). The most prominent confusion pattern involved sadness being misidentified as disgust, with mean error rates of 0.85 (SD = 1.38) in younger adults and 1.19 (SD = 1.33) in older adults. Fear was frequently confused with surprise in both groups (0.86 ± 1.42 and 1.19 ± 1.66, respectively), and anger was consistently misattributed as surprise (0.93 ± 1.68 and 1.14 ± 1.80). Descriptively, young men showed a tendency toward elevated bidirectional anger-disgust confusion compared to young women; however, this pattern did not reach statistical significance after correction for within-participant clustering (*p* = .077) and is not reflected in the normative stratification. Older adults (60+) exhibited two distinctive error patterns that were substantially amplified relative to younger adults: misattribution of fear as anger (1.38 ± 1.83 vs. 0.23 ± 0.70) and of surprise as joy (1.00 ± 1.92 vs. 0.37 ± 0.72).

#### Reaction time for emotions

Data for response times on the FER task are presented in Table 3. Overall, results revealed fastest processing for joy, slowest responses for anger recognition, and substantial age-related slowing across all emotions after 50 years of age.

**Table 3:** Response times (in milliseconds) on the FER (IDEMO) task

Group	18–49	50+
n	108	47
Men (age)	48 (31.96 ± 7.23)	17 (61.24 ± 3.99)
Women (age)	60 (34.22 ± 8.20)	30 (56.30 ± 5.39)
RT_Anger	2383.59 ± 1309.01	3327.00 ± 1588.07
RT_Disgust	1700.94 ± 980.65	2112.54 ± 1033.44
RT_Joy	1177.86 ± 377.86	1601.57 ± 627.83
RT_Fear	1937.56 ± 908.00	2745.42 ± 1351.91
RT_Surprise	1852.92 ± 824.30	2497.14 ± 1230.27
RT_Sadness	2098.87 ± 1100.52	2798.20 ± 1268.84

Two normative groups were established based on preliminary age effect analyses: adults aged 18–49 years (*n* = 108) and adults aged 50 years and older (*n* = 47). Across both groups, joy elicited the shortest response times (1177.86 ms and 1601.57 ms, respectively), whereas anger required the longest processing time (2383.59 ms and 3327.00 ms). The older normative group showed consistently longer response times across all emotions compared to younger adults, with increases ranging from 24.2% for disgust to 41.7% for fear. Values are presented as mean ± standard deviation; age is reported in years. Percentile-based normative data are provided in Additional File 4

A three-way mixed-model ANOVA examined the effects of age group (five levels: 18–29, 30–39, 40–49, 50–59, 60+), sex, and emotion on response times. Mauchly’s test indicated a violation of sphericity for the emotion factor, W = 0.609, χ² (14) = 75.41, *p* < .001; degrees of freedom for all within-subjects effects were therefore corrected using the Greenhouse-Geisser adjustment (ε = 0.837). Results revealed a significant main effect of emotion [F(4.19, 728.51) = 30.47, *p* < .001, ηp² = 0.149, 90% CI [0.114, 0.182], ωp² = 0.137], indicating large differences in processing speed across emotion categories. A significant main effect of age group was also observed [F(4, 870) = 24.23, *p* < .001, ηp² = 0.100, 90% CI [0.069, 0.130], ωp² = 0.091], reflecting a medium-sized age-related effect on response latencies. The age × sex interaction reached significance [F(4, 870) = 2.51, *p* = .041, ηp² = 0.011, 90% CI [0.001, 0.022], ωp² = 0.007], indicating a small but reliable moderating effect of sex on age-related slowing. The main effect of sex was not significant [F(1, 870) = 1.13, *p* = .289, ηp² = 0.001, ωp² < 0.001], nor were the age × emotion [F(16.75, 728.51) = 0.61, *p* = .886, ηp² = 0.014, ωp² < 0.001], sex × emotion [F(4.19, 728.51) = 0.47, *p* = .768, ηp² = 0.003, ωp² < 0.001], or three-way interactions [F(16.75, 728.51) = 0.50, *p* = .954, ηp² = 0.011, ωp² < 0.001] significant.

Post-hoc Tukey HSD tests on the ten age × sex subgroups revealed a complex pattern primarily driven by age. Participants aged 50–59 of both sexes showed significantly slower response times compared to younger groups: women aged 50–59 (F_50–59) were slower than men aged 18–29 (*p* = .001, d = 0.51), men aged 30–39 (*p* < .001, d = 0.72), men aged 40–49 (*p* = .038, d = 0.50), and women aged 18–29 through 40–49 (all *p* < .05, d ranging from 0.36 to 0.67). Men aged 50–59 (H_50–59) demonstrated the longest response times overall, differing from men aged 18–29 (*p* < .001, d = 1.44), men aged 30–39 (*p* < .001, d = 1.52), women aged 18–29 (*p* < .001, d = 1.35), and women aged 30–39 (*p* < .001, d = 1.34), all reflecting large effect sizes. Notably, men aged 60 + responded faster than men aged 50–59 (*p* = .014, d = 0.80), a medium-to-large effect. Younger participants (18–49 years) showed no significant differences across sex (all *p* > .05, all d < 0.35). Based on this pattern, two normative groups were established for emotion-based response times: participants under 50 years of age and participants aged 50 years and older.

Across both groups, response times were shortest for joy recognition (1177.86 ± 377.86 ms and 1601.57 ± 627.83 ms, respectively), consistent with the ceiling effects observed for accuracy on this emotion. In contrast, anger elicited the longest response times in both groups (2383.59 ± 1309.01 ms in the 18–49 (11) group and 3327.00 ± 1588.07 ms in the 50 + group), followed by sadness and fear. The older normative group (50+) exhibited substantially longer mean response times across all emotions compared to younger adults (18–49), with increases of 39.6% for anger, 36.0% for joy, 41.7% for fear, 34.8% for surprise, and 33.3% for sadness, suggesting marked age-related slowing in facial emotion processing speed after 50 years of age. Notably, response time distributions showed substantial inter-individual variability, as reflected by standard deviations often exceeding 40% of mean values, particularly for anger in both groups.

#### Reaction times for intensities

Data for response times across emotional intensity levels on the FER (IDEMO) task are presented in Table 4. Overall, results revealed a facilitation effect of higher emotional intensity on response speed, and a nonlinear age-related pattern with the 50–59 age group showing the longest response times across most intensity levels.

**Table 4:** Response times (ms) on the FER (IDEMO) task by emotional intensity level

Group	18–39	40–49	50–59	60+
n	83	25	26	21
Men (age)	42 (30.33 ± 6.04)	6 (43.33 ± 3.83)	4 (56.50 ± 3.11)	13 (62.69 ± 3.01)
Women (age)	41 (29.85 ± 5.64)	19 (43.63 ± 3.59)	22 (53.64 ± 3.08)	8 (63.62 ± 2.88)
Reaction_Time(1)	2120.89 ± 971.68	2476.34 ± 1255.18	3024.45 ± 1416.61	2752.76 ± 1296.85
Reaction_Time(2)	1943.51 ± 1115.64	2063.73 ± 1012.09	2688.88 ± 1126.89	2365.05 ± 1252.43
Reaction_Time(3)	1878.63 ± 834.98	2176.88 ± 894.30	2909.72 ± 1501.04	2391.94 ± 1087.23
Reaction_Time(4)	1795.15 ± 810.14	2307.43 ± 1243.41	2669.33 ± 1206.86	2443.66 ± 1077.32
Reaction_Time(5)	1693.98 ± 776.63	2046.48 ± 849.12	2646.35 ± 1107.48	2116.86 ± 761.96
Reaction_Time(6)	1782.86 ± 859.04	2114.01 ± 1192.49	2625.58 ± 1166.67	2202.43 ± 926.70
Reaction_Time(7)	1810.04 ± 1036.22	1972.16 ± 922.07	2965.59 ± 1674.20	2390.15 ± 1566.46
Reaction_Time(8)	1753.64 ± 955.77	1975.99 ± 1076.67	2510.48 ± 1091.09	2041.85 ± 1002.03
Reaction_Time(9)	1536.53 ± 500.48	1866.65 ± 1079.04	2391.72 ± 1152.73	2017.80 ± 750.57
Reaction_Time(10)	1571.32 ± 663.50	1667.42 ± 545.19	2406.53 ± 1052.97	1997.97 ± 770.11

Four normative groups were established based on age: young adults (18–39 years), middle-aged adults (40–49 years), older middle-aged adults (50–59 years), and older adults (60 + years). Reaction_Time (1) corresponds to the lowest emotional intensity and Reaction_Time (10) to the highest. Response times decreased with increasing intensity across all groups, reflecting the facilitation effect of emotional salience on recognition speed. Notably, the 50–59 age group demonstrated the longest response times across most intensity levels, followed by the 60 + group. This descriptive pattern departs from a simple linear age trajectory, though its interpretation is constrained by modest sample sizes (*n* = 26 and *n* = 21, respectively). Values are presented as mean ± SD in milliseconds. Percentile-based normative data are provided in Additional File 5

A three-way mixed-model ANOVA examined the effects of age group (5 levels: 18–29, 30–39, 40–49, 50–59, 60+), sex, and intensity level (10 levels, treated categorically) on response times. Mauchly’s test indicated a violation of sphericity for the intensity factor, W = 0.283, χ² (44) = 190.62, *p* < .001; degrees of freedom for all within-subjects effects were therefore corrected using the Greenhouse-Geisser adjustment (ε = 0.779). Results revealed a significant main effect of age group [F(4, 1450) = 43.70, *p* < .001, ηp² = 0.108, 90% CI [0.081, 0.131], ωp² = 0.099], representing a medium-sized effect, and a significant main effect of intensity [F(7.01, 1129.25) = 5.37, *p* < .001, ηp² = 0.032, 90% CI [0.013, 0.043], ωp² = 0.025], representing a small effect. A significant age × sex interaction was also observed [F(4, 1450) = 4.42, *p* = .001, ηp² = 0.012, 90% CI [0.002, 0.021], ωp² = 0.009]. The main effect of sex was not significant [F(1, 1450) = 2.19, *p* = .139, ηp² = 0.002, ωp² < 0.001], nor were the age × intensity [F(28.04, 1129.25) = 0.27, *p* = 1.000, ηp² = 0.007, ωp² < 0.001], sex × intensity [F(7.01, 1129.25) = 0.38, *p* = .914, ηp² = 0.002, ωp² < 0.001], or three-way interactions [F(28.04, 1129.25) = 0.50, *p* = .987, ηp² = 0.012, ωp² < 0.001] significant. A complementary ANCOVA treating intensity as a continuous covariate confirmed significant effects of age group [F(4, 1539) = 45.14, *p* < .001, ηp² = 0.105, 90% CI [0.079, 0.128], ωp² = 0.102], the age × sex interaction [F(4, 1539) = 4.57, *p* = .001, ηp² = 0.012, 90% CI [0.002, 0.020], ωp² = 0.009], and a linear effect of intensity [F(1, 1539) = 40.06, *p* < .001, ηp² = 0.025, 90% CI [0.013, 0.040], ωp² = 0.025], whereas the main effect of sex remained non-significant [F(1, 1539) = 2.26, *p* = .133, ηp² = 0.001, ωp² < 0.001].

Post-hoc Tukey HSD tests on age groups revealed a complex pattern of differences. The youngest groups (18–29 and 30–39) did not differ significantly from each other (adjusted *p* = .262, d = 0.16, negligible), nor did 18–29 differ from 40 to 49 (adjusted *p* = .099, d = 0.22, small). However, both young groups differed significantly from 50 to 59 (18–29 vs. 50–59: adjusted *p* < .001, d = 0.80, 95% CI [0.63, 0.96]; 30–39 vs. 50–59: adjusted *p* < .001, d = 0.91, 95% CI [0.75, 1.07]) and 60+ (18–29 vs. 60+: adjusted *p* < .001, d = 0.44, 95% CI [0.27, 0.61]; 30–39 vs. 60+: adjusted *p* < .001, d = 0.57, 95% CI [0.40, 0.73]). The 30–39 group differed from 40 to 49 (adjusted *p* < .001, d = 0.36, 95% CI [0.20, 0.51]), and 40–49 differed from 50 to 59 (adjusted *p* < .001, d = 0.53, 95% CI [0.36, 0.71]) but not from 60+ (adjusted *p* = .186, d = 0.19, negligible). Notably, 50–59 and 60 + differed significantly (adjusted *p* < .001, d = 0.35, 95% CI [0.16, 0.53]), with the 50–59 group showing longer response times.

Post-hoc tests on intensity levels indicated that the lowest intensity (level 10) was associated with significantly longer response times compared to levels 14 (adjusted *p* = .007, d = 0.42), 15 (adjusted *p* = .045, d = 0.34), 17 (adjusted *p* = .005, d = 0.41), 18 (adjusted *p* < .001, d = 0.60), and 19 (adjusted *p* < .001, d = 0.62), with effect sizes ranging from small to medium. Additionally, level 12 differed significantly from level 19 (adjusted *p* = .041, d = 0.41).

Although the age × sex interaction was significant, post-hoc comparisons on the ten sex × age subgroups indicated that sex differences were limited in scope. No significant sex differences were observed within the 18–29 (adjusted *p* = .264, d = 0.31), 30–39 (adjusted *p* = 1.000, d = 0.02), 40–49 (adjusted *p* = .965, d = 0.19), or 60+ (adjusted *p* = .999, d = 0.10) age groups. A significant sex difference emerged only within the 50–59 group, with men showing longer response times than women (adjusted *p* = .027, d = 0.46, 95% CI [0.13, 0.80]). Men aged 50–59 displayed the longest response times overall, differing significantly from nearly all other subgroups, with large effect sizes relative to young adults (d = 1.48–1.69 compared to women and men aged 18–39).

Based on these results, four normative groups were established for intensity-based response times: young adults (18–39), middle-aged adults (40–49), older middle-aged adults (50–59), and older adults (60+). Although a sex difference was observed within the 50–59 group, separate sex-based norms were not created to maintain clinical practicality; clinicians should note that men in this age range may exhibit slightly longer response times.

Response times decreased as emotional intensity increased across all groups, consistent with the expected facilitation effect of higher intensity on emotion recognition. For the lowest intensity level (level 10), mean response times were 2120.89 ms (SD = 971.68), 2476.34 ms (SD = 1255.18), 3024.45 ms (SD = 1416.61), and 2752.76 ms (SD = 1296.85) for young adults, middle-aged adults, older middle-aged adults, and older adults, respectively. At the highest intensity level (level 19), response times decreased to 1571.32 ms (SD = 663.50), 1667.42 ms (SD = 545.19), 2406.53 ms (SD = 1052.97), and 1997.97 ms (SD = 770.11) for the same groups. Notably, the 50–59 age group consistently exhibited the longest response times across all intensity levels, even exceeding those of the 60 + group. Descriptively, this pattern is consistent with the significant age × sex interaction observed in the ANOVA, which was statistically driven by men aged 50–59 demonstrating particularly prolonged response times. However, given the limited clinical utility of sex-stratified norms within this narrow age band, a unified normative group was retained for the 50–59 cohort with clinicians advised that men in this range may exhibit slightly longer latencies. Substantial inter-individual variability was observed across all groups, as reflected by relatively large standard deviations, particularly at lower intensity levels and within the 50–59 age group.

#### Reading the mind in the eyes

Data for accuracy and response time on the Reading the Mind in the Eyes Test are presented in Table 5. Overall, results indicated an age-related decline in mental state recognition, with lower accuracy scores in older adults. Response time analyses revealed a non-linear age pattern, with middle-aged adults (50–59 years) exhibiting the longest latencies.

**Table 5:** Scores and response times (ms) on the Reading the Mind in the Eyes Test

Measure	Score		Reaction Time		
Group	18–59	60+	18–49	50–59	60+
n	153	22	125	28	22
Men (age)	63 (32.57 ± 9.81)	12 (62.67 ± 3.20)	58 (30.59 ± 7.30)	5 (55.60 ± 3.36)	12 (62.67 ± 3.20)
Women (age)	90 (39.09 ± 11.43)	10 (63.60 ± 2.80)	67 (34.10 ± 8.61)	23 (53.61 ± 3.19)	10 (63.60 ± 2.80)
Values	21.25 ± 3.12	18.91 ± 2.96	6617.80 ± 2364.79	8709.23 ± 3818.18	5699.94 ± 1987.29

For accuracy, participants were stratified into younger (18–59 years) and older (60 + years) adults, revealing a decline in mental state recognition with advancing age. For response times, three age groups were established based on significant post-hoc differences: young adults (18–49), middle-aged adults (50–59), and older adults (60+). The 50–59 group exhibited the longest response latencies. Sample sizes, sex distribution with corresponding mean ages (± SD), and descriptive statistics (mean ± SD) are provided for each measure. Percentile-based normative data are provided in Additional Files 6 and 7

A two-way ANOVA examined the effects of age group (five levels: 18–29, 30–39, 40–49, 50–59, 60+) and sex on RMET accuracy scores. Results revealed a significant main effect of age group [F(4, 165) = 3.33, *p* = .012, ηp² = 0.075, 90% CI [0.008, 0.130], ωp² = 0.051], representing a medium-sized effect. The main effect of sex was not significant [F(1, 165) = 0.15, *p* = .698, ηp² = 0.001, ωp² < 0.001]. The age × sex interaction reached significance [F(4, 165) = 2.53, *p* = .043, ηp² = 0.058, 90% CI [0.001, 0.107], ωp² = 0.034]; however, cell sizes for older male participants were insufficient (*n* = 9) to establish sex-specific norms, and this interaction pattern warrants replication in larger samples. Post-hoc Tukey HSD tests indicated that older adults (60+) scored significantly lower than both the 18–29 group (adjusted *p* = .007, d = 1.01, 95% CI [0.48, 1.53]) and the 30–39 group (adjusted *p* = .023, d = 0.79, 95% CI [0.27, 1.32]), representing large and medium effect sizes, respectively. No other pairwise comparisons reached significance (all d < 0.59, all *p* > .10). Accordingly, two normative groups were established for RMET accuracy: adults aged 18–59 years and older adults aged 60 years and above. Mean accuracy was 21.25 (SD = 3.12) in the younger group and 18.91 (SD = 2.96) in the older group.

For RMET response times, the two-way ANOVA revealed a significant main effect of age group [F(4, 165) = 6.32, *p* < .001, ηp² = 0.133, 90% CI [0.047, 0.202], ωp² = 0.108], representing a medium-sized effect. The main effect of sex [F(1, 165) = 0.54, *p* = .466, ηp² = 0.003, ωp² < 0.001] and the age × sex interaction [F(4, 165) = 0.88, *p* = .479, ηp² = 0.021, ωp² < 0.001] were not significant. Post-hoc Tukey HSD tests indicated that the 50–59 group exhibited significantly longer response times than the 30–39 group (adjusted *p* < .001, d = 0.94, 95% CI [0.45, 1.43]), the 40–49 group (adjusted *p* = .048, d = 0.63, 95% CI [0.09, 1.18]), and the 60 + group (adjusted *p* < .001, d = 0.96, 95% CI [0.37, 1.54]), all representing medium-to-large effect sizes. No other pairwise comparisons reached significance, though the 18–29 vs. 60 + contrast approached a medium effect (d = 0.59, 95% CI [0.08, 1.10], *p* = .194). Accordingly, three normative groups were established for RMET response times: adults aged 18–49, adults aged 50–59, and older adults aged 60 years and above.

Older adults aged 60 years and above (*n* = 22) showed the shortest mean response time of 5699.94 ms (SD = 1987.29). This pattern departs from a simple linear age-related slowing trajectory; however, given the modest sample sizes in the 50–59 (*n* = 28) and 60+ (*n* = 22) groups, this observation should be considered preliminary and interpreted with caution.

#### Theory of mind

Data for the Theory of Mind task are presented in Table 6. Overall, results revealed ceiling effects for detection and high accuracy for memory and factual comprehension, with greater variability observed for false belief attribution, inference, and empathy components. Performance was higher on first-order (M = 9.26) compared to second-order tasks (M = 6.10), consistent with the increased cognitive demand of higher-order mentalizing.

**Table 6:** Theory of Mind (ToM) performance by order of inference and cognitive dimension

Group	All adults (18–69)
n	129
Men (age)	52 (38.13 ± 13.52)
Women (age)	77 (41.56 ± 12.77)
Order	
First-Order	9.26 ± 0.96
Second-Order	6.1 ± 0.97
Faux-pas	12.88 ± 1.18
Total	28.24 ± 1.98
Dimension	
Memory	5.77 ± 0.48
Inference	5.51 ± 0.77
False Belief	4.49 ± 1.07
Factual	4.95 ± 0.3
Detection	2.0 ± 0.0
Identification	1.93 ± 0.28
Non intentionality	1.88 ± 0.35
Empathy	1.71 ± 0.5

Order-based analysis revealed decreasing performance from first-order (9.26 ± 0.96) to second-order tasks (6.10 ± 0.97), reflecting the increased cognitive demand of recursive mentalizing. Faux-pas recognition yielded the highest subscore (12.88 ± 1.18), contributing substantially to the total score (28.24 ± 1.98). Dimensional analysis indicated ceiling effects for detection (2.00 ± 0.00) and near-ceiling performance for memory (5.77 ± 0.48) and factual comprehension (4.95 ± 0.30). Greater interindividual variability was observed for false belief attribution (4.49 ± 1.07), inference (5.51 ± 0.77), and empathy (1.71 ± 0.50), suggesting these dimensions may be more sensitive to individual differences and potentially to clinical impairment. Data are presented as mean ± SD. In group labels, numeric ranges (e.g., 18–29) indicate age in years. Percentile-based normative data are provided in Additional File 8 for both order and dimension

Two separate mixed-model ANOVAs were conducted to examine effects of age group (18–29, 30–39, 40–49, 50–59, 60 + years) and sex on Theory of Mind performance, with either order of inference (first-order, second-order, faux pas) or cognitive dimension (memory, inference, false belief, factual, detection, identification, non-intentionality, empathy) as the within-subjects factor.

For the order-based analysis, Mauchly’s test indicated that the sphericity assumption was met, W = 0.968, χ² (2) = 4.15, *p* = .125. Further results revealed a significant main effect of order [F(2, 4227) = 85.55, *p* < .001, ηp² = 0.039, 90% CI [0.030, 0.049], ωp² = 0.038], reflecting small-to-medium differential performance across inference levels. Post-hoc Tukey HSD comparisons indicated that first-order performance was significantly higher than both faux pas (mean difference = 0.078, *p* < .001, d = 0.26, 95% CI [0.19, 0.33]) and second-order tasks (mean difference = 0.080, *p* < .001, d = 0.23, 95% CI [0.15, 0.31]), both reflecting small effect sizes, while faux pas performance was significantly higher than second-order tasks (mean difference = 0.158, *p* < .001, d = 0.52, 95% CI [0.44, 0.60]), a medium effect. A marginally significant main effect of age group was observed [F(4, 4227) = 2.38, *p* = .050, ηp² = 0.002, ωp² = 0.001]; however, the effect size was negligible, and post-hoc Tukey HSD comparisons revealed no significant pairwise differences between any age groups (all *p* > .23, all d < 0.10). The main effect of sex was not significant [F(1, 4227) = 0.82, *p* = .366, ηp² < 0.001, ωp² < 0.001], nor were any of the interactions: order × age [F(8, 4227) = 1.16, *p* = .317, ηp² = 0.002, ωp² < 0.001], order × sex [F(2, 4227) = 0.50, *p* = .607, ηp² < 0.001, ωp² < 0.001], age × sex [F(4, 4227) = 0.35, *p* = .847, ηp² < 0.001, ωp² < 0.001], or the three-way interaction [F(8, 4227) = 1.49, *p* = .157, ηp² = 0.003, ωp² = 0.001].

For the dimension-based analysis, Mauchly’s test indicated a significant violation of sphericity, W = 0.027, χ² (27) = 454.44, *p* < .001; degrees of freedom for all within-subjects effects were therefore corrected using the Greenhouse-Geisser adjustment (ε = 0.542). Results revealed a significant main effect of cognitive dimension [F(3.80, 2265.33) = 130.10, *p* < .001, ηp² = 0.179, 90% CI [0.160, 0.195], ωp² = 0.175], a large effect indicating substantial differential performance across ToM components. Post-hoc Tukey HSD comparisons revealed a hierarchical pattern: false belief scores were significantly lower than all other dimensions (all *p* < .001), with large effect sizes relative to memory (d = 0.93), factual comprehension (d = 1.04), detection (d = 0.94), and identification (d = 0.83), and small-to-medium effects relative to inference (d = 0.38) and non-intentionality (d = 0.74). Inference scores were significantly lower than memory (d = 0.63, medium), factual (d = 0.78, medium), detection (d = 0.73, medium), identification (d = 0.59, medium), and non-intentionality (d = 0.48, small), all *p* < .001, but significantly higher than false belief (*p* < .001). Empathy scores differed significantly from all other dimensions (all *p* < .05), with medium effects relative to factual comprehension (d = 0.67) and detection (d = 0.60), and small effects relative to identification (d = 0.40), memory (d = 0.44), inference (d = 0.21), and non-intentionality (d = 0.28). In contrast, memory, factual, detection, identification, and non-intentionality scores did not significantly differ from one another (all *p* > .20, all d < 0.20). A significant main effect of age group was also observed [F(4, 4177) = 2.77, *p* = .026, ηp² = 0.003, ωp² = 0.002]; however, the effect size was negligible, and post-hoc Tukey HSD comparisons again revealed no significant pairwise differences between any age groups (all *p* > .23, all d < 0.10). The main effect of sex was not significant [F(1, 4177) = 0.95, *p* = .330, ηp² < 0.001, ωp² < 0.001], nor were any of the interactions: dimension × age [F(15.19, 2265.33) = 0.89, *p* = .575, ηp² = 0.006, ωp² < 0.001], dimension × sex [F(3.80, 2265.33) = 0.86, *p* = .485, ηp² = 0.001, ωp² < 0.001], age × sex [F(4, 4177) = 0.40, *p* = .807, ηp² < 0.001, ωp² < 0.001], or the three-way interaction [F(15.19, 2265.33) = 0.81, *p* = .668, ηp² = 0.005, ωp² < 0.001].

Given that the significant main effects of age group did not translate into any significant pairwise differences between specific age groups in post-hoc analyses, and considering both the negligible effect sizes (all ηp² < 0.003, all d < 0.10) and the absence of significant sex effects and interactions, normative data were computed across a single adult group (18–99 years) for both order-based and dimension-based scores.

When analyzed by order of inference, participants achieved mean scores of 9.26 (SD = 0.96) for first-order tasks, 6.10 (SD = 0.97) for second-order tasks, and 12.88 (SD = 1.18) for faux-pas recognition, yielding a total score of 28.24 (SD = 1.98). The dimensional analysis revealed high performance on memory (M = 5.77, SD = 0.48) and factual comprehension (M = 4.95, SD = 0.30), with ceiling effects observed for detection (M = 2.00, SD = 0.00). Greater variability was observed for false belief attribution (M = 4.49, SD = 1.07), inference (M = 5.51, SD = 0.77), and empathy (M = 1.71, SD = 0.50). Identification of mental states (M = 1.93, SD = 0.28) and recognition of non-intentionality (M = 1.88, SD = 0.35) showed relatively low variability across participants.

## Discussion

The present study introduces a multilevel interpretive framework for the Social Cognition Battery (BCS) that moves beyond conventional aggregate scoring to exploit the full dimensionality of data generated during social cognitive assessment. By establishing empirically derived normative data for emotion-specific accuracy, intensity-dependent performance profiles, systematic misattribution patterns, response latencies, and theory of mind subcomponents, this approach addresses several limitations that have constrained the clinical utility of social cognition measures using the BCS.

A central finding of this work is that clinically meaningful variability resides at the level of specific subcomponents rather than global scores, as noted by previous works [79, 80]. On the facial emotion recognition task, total accuracy scores obscured substantial heterogeneity across emotions: while joy and disgust demonstrated ceiling effects across all age groups, anger and fear showed marked variability and age-related decline; consistently with available data in the literature [91–95]. This dissociation carries diagnostic implications, as a patient presenting with selective impairment in anger and fear recognition (but preserved joy and disgust identification) would obtain a moderately reduced total score indistinguishable from a patient with uniformly mild deficits across all emotions. Yet these two profiles likely reflect distinct underlying mechanisms [96–98] and may respond differently to intervention [99, 100].

The integration of response time data represents a particularly valuable extension of standard scoring practices. Processing speed provides information about cognitive efficiency that accuracy alone cannot capture [101, 102], a distinction with important clinical ramifications. High-functioning individuals with social cognitive difficulties (including adults with autism spectrum conditions) may achieve near-normal accuracy through compensatory effortful processing, a phenomenon sometimes termed “camouflaging” [47, 48]. It has been hypothesized that such individuals, while undetected by accuracy-based screening, could be identified through prolonged response latencies [41, 42, 103]. Although the present normative data do not directly test this compensation hypothesis, they provide the reference distributions against which such accuracy–latency dissociations could be evaluated in future clinical studies.

Our data revealed that response times varied systematically with both emotion category and stimulus intensity: joy elicited the fastest responses across all groups, consistent with its ceiling-level accuracy, whereas anger required substantially longer processing time despite intermediate accuracy levels. This pattern suggests that anger recognition engages more deliberative processing even in neurotypical adults [104, 105], a baseline against which clinical slowing can be evaluated. The unexpected finding that adults aged 50–59 exhibited longer latencies than those over 60 is noteworthy but must be interpreted cautiously given the limited sample sizes in both groups (*n* = 28 and *n* = 22, respectively). Although this pattern could reflect qualitative shifts in processing strategy across the lifespan, such as increased deliberative effort in middle-aged adults or reliance on faster heuristic strategies in older adults [106, 107]. Regardless of the underlying mechanism, this observation illustrates the added value of response time data, which may reveal age-related patterns that remain invisible in accuracy scores alone [80, 108].

The systematic quantification of emotional confusion patterns through misattribution matrices offers a third dimension of clinical information. Error patterns are routinely observed during assessment but rarely formalized into interpretive frameworks, leaving their analysis to informal clinical judgment [25]. Our data revealed that certain confusions (e.g. sadness misidentified as disgust, fear as surprise) occurred systematically across all demographic groups, likely reflecting perceptual similarities in facial muscle configurations (e.g., widened eyes for both fear and surprise; downturned mouth for both sadness and disgust; [109–111]). Other patterns showed demographic specificity: descriptively, young men showed a tendency toward elevated bidirectional confusion between anger and disgust compared to young women, though this effect did not reach statistical significance after correction for within-participant clustering (*p* = .077) and requires replication in larger samples. This pattern would be consistent with documented gender differences in the processing of negative facial affects [112, 113]. Older adults demonstrated distinctive fear-anger confusions largely absent in younger groups, in line with meta-analytic evidence on age-related emotion recognition decline [78, 80]. These systematic error signatures may inform mechanistic understanding of social cognitive deficits [28, 114]. For instance, elevated fear-anger confusion in a clinical population might suggest difficulty discriminating threat-related emotions specifically, whereas broad confusion across multiple emotion pairs might indicate more general perceptual or conceptual impairment [30, 51].

The heterogeneous effects of age across social cognition components warrant theoretical consideration. Facial emotion recognition showed clear age-related decline after 60 years, consistent with meta-analytic evidence documenting reduced accuracy for negative emotions (particularly anger, fear, and sadness) in older adulthood [79, 80]. Importantly, this decline was most pronounced at lower intensity levels, where emotional expressions are subtle and ambiguous. This pattern aligns with previous findings showing that age differences are magnified when expressions are less prototypical [115, 116], an effect which presents ecological validity: naturalistic emotional expressions rarely achieve the prototypical intensity of standardized stimuli, suggesting that laboratory-based ceiling effects may underestimate real-world difficulties experienced by older adults [117, 118].

In contrast, theory of mind performance showed no significant age-related decline when examined, despite omnibus effects reaching statistical significance. This pattern aligns with proposals that age-related changes in mentalizing may be partially mediated by executive function demands rather than reflecting deterioration of social cognitive representations per se [85, 119, 120]. Indeed, evidence suggests that ToM competence may remain intact in older adults when task demands are reduced, implicating executive function decline as the primary source of age-related ToM difficulties [121]. The ToM tasks employed here impose relatively modest working memory and inhibitory control demands compared to more complex paradigms; age effects might emerge with tasks requiring integration of multiple mental states or rapid online updating of social inferences [122–124].

Sex differences, though statistically modest, showed theoretically interesting specificity. The female advantage in intensity-based accuracy and the marked sex difference in anger-disgust confusion among young adults converge with literature suggesting that women demonstrate enhanced sensitivity to subtle emotional cues, particularly for threat-related expressions [82, 125–127]. Although sex × age interactions reached significance for response time measures, no such interactions were confirmed for misattribution patterns after appropriate correction for within-participant dependency (all *p* > .12). The descriptive attenuation of sex differences with age observed in confusion matrices may reflect convergence through differential trajectories of decline or compensatory strategy development, but this pattern requires confirmation in larger samples. These patterns align with previous findings showing that sex differences in emotion recognition are not uniform across the lifespan [128, 129] and underscore the value of data-driven stratification: imposing uniform sex-based norms across all measures would have been inappropriate given the heterogeneity of sex effects across tasks and age groups.

The differential patterns observed across facial emotion recognition, eye-based mental state inference, and theory of mind reasoning are consistent with multidimensional models of social cognition [86, 130], though they do not constitute direct evidence for such models. Age affected FER and RMET accuracy while sparing ToM; response time patterns diverged across tasks; and the demographic stratifications derived empirically differed for each measure. However, these dissociations should be interpreted cautiously, as they may partly reflect task-specific psychometric properties (e.g. differences in ceiling effects, scoring distributions, and item difficulty) as well as differential exclusion patterns across subtests (e.g., 155 participants for FER vs. 129 for ToM). Thus, the observed pattern aligns with prior meta-analytic evidence showing differential age trajectories for emotion recognition versus theory of mind tasks [79, 80] and is compatible with distinctions between “hot” affective components of social cognition and “cold” cognitive-inferential components [36]. Neuroimaging and lesion studies demonstrating separable neural substrates for affective and cognitive theory of mind [62, 63] provide independent grounds for expecting such dissociations, but the present normative data alone cannot adjudicate between psychometric and neurocognitive explanations for the divergent profiles observed here. The finding that intensity level modulated both accuracy and response times (with ambiguous expressions revealing greater individual differences and age effects) suggests that stimulus characteristics interact with person-level factors in determining social cognitive performance, a pattern documented in normative studies using morphed expression paradigms [27, 116] and often neglected when standardized stimuli are treated as interchangeable. Establishing whether the dissociations documented here reflect separable cognitive mechanisms will require confirmatory approaches, including convergent multi-method assessment and direct comparison across clinical populations with theoretically predicted dissociation profiles.

The interpretive framework proposed here has the potential to enhance clinical practice in several ways, although these applications remain to be validated in clinical populations. First, by providing normative benchmarks at the level of specific emotions, intensity gradients, and error patterns, the framework could facilitate the identification of selective impairments and, in turn, inform individualized rehabilitation planning. For instance, a patient presenting with specific difficulty recognizing fear might benefit from targeted training on threat-related cues, whereas one with generalized slowing across emotions might require interventions addressing processing efficiency. Second, the incorporation of response time percentile norms offers a potential means of detecting compensatory strategies that mask underlying difficulties, information that could prove valuable for prognostic formulation and outcome monitoring. Third, the formalization of confusion patterns may provide a basis for mechanistic hypotheses that could guide further assessment and intervention. Critically, whether these finer-grained normative distinctions translate into improved diagnostic sensitivity, differential diagnostic value, or treatment planning over conventional summary scores is an empirical question that requires systematic investigation across clinical populations with known social cognitive impairments.

Future research should examine whether the multilevel profiles derived from this framework differentiate among clinical populations with known social cognitive impairments. Preliminary hypotheses can be generated: frontotemporal dementia might be characterized by disproportionate impairment in negative emotion recognition with preserved ToM initially; autism spectrum conditions might show preserved accuracy with prolonged response times reflecting effortful compensation; schizophrenia might demonstrate elevated confusion across multiple emotion pairs reflecting broader perceptual-cognitive disorganization. Testing such hypotheses would establish the diagnostic utility of this approach and potentially inform differential diagnosis.

## Limitations

Several limitations constrain interpretation of these findings. First, the normative sample, though adequate for establishing preliminary norms, included relatively few older adults and showed sex imbalances within certain age strata. The significant age × sex interactions observed for several measures could not be fully characterized due to small cell sizes, and sex-stratified norms for older adults await replication in larger samples. Moreover, ceiling effects for joy and disgust recognition and for several ToM dimensions (notably detection, with zero variance) limit the sensitivity of these measures to subtle impairments. Future revisions of the battery might incorporate more challenging items, perhaps expressions blending multiple emotions or requiring discrimination among perceptually similar mental states, to extend the dynamic range of assessment.

On another hand, the generalizability of these norms to non-French-speaking populations remains untested. Social cognition is shaped by cultural display rules, linguistic categories for emotions, and socialization practices that vary across cultural contexts. While the basic emotions assessed here are generally considered universal, recognition thresholds and confusion patterns may differ cross-culturally. Finally, the present study focused exclusively on healthy adults; the clinical validity of this multilevel framework (i.e. its sensitivity and specificity for detecting impairments in neurological and psychiatric populations) requires systematic investigation across diagnostic groups.

## Conclusion

In conclusion, the multilevel interpretive methodology proposed here extends the BCS beyond summary scores by providing a finer-grained normative framework that captures the multidimensional structure of social cognitive performance. By accounting for the inherent complexity of social cognition (i.e. its multiple components, sensitivity to stimulus characteristics, and dependence on processing efficiency as well as accuracy) this approach has the potential to improve future clinical interpretation by enabling more precise characterization of individual performance profiles. However, the present findings are derived from a healthy normative sample, and the clinical utility of this framework, including its sensitivity for detecting impairments, its capacity to inform differential diagnosis, and its relevance for individualized rehabilitation planning, remains to be established through systematic validation in neurological and psychiatric populations.

## Supplementary Information

Supplementary Material 1.

Supplementary Material 2.

Supplementary Material 3.

Supplementary Material 4.

Supplementary Material 5.

Supplementary Material 6.

Supplementary Material 7.

Supplementary Material 8.

## Data Availability

The raw data used in this study are openly available in the online archives of the Groupe de Réflexion sur les Evaluations Cognitives (GRECO; https://www.site-greco.net). Percentiles and norms are available alongside the present manuscript.
